# Robustness of the Tariff method for diagnosing verbal autopsies: impact of additional site data on the relationship between symptom and cause

**DOI:** 10.1186/s12874-019-0877-7

**Published:** 2019-12-09

**Authors:** Hafizur Rahman Chowdhury, Abraham D. Flaxman, Jonathan C. Joseph, Riley H. Hazard, Nurul Alam, Ian Douglas Riley, Alan D. Lopez

**Affiliations:** 1 School of Population and Global Health, University of Melbourne, Parkville, VIC Australia; 2 Institute for Health Metrics and Evaluation, University of Washington, Seattle, Washington, USA; 3 International Center for Diarrhoeal Disease Research, Dhaka, Bangladesh

**Keywords:** Verbal autopsy, Tariff, Symptom, Cause of death, Gold standard

## Abstract

**Background:**

Verbal autopsy (VA) is increasingly being considered as a cost-effective method to improve cause of death information in countries with low quality vital registration. VA algorithms that use empirical data have an advantage over expert derived algorithms in that they use responses to the VA instrument as a reference instead of physician opinion. It is unclear how stable these data driven algorithms, such as the Tariff 2.0 method, are to cultural and epidemiological variations in populations where they might be employed.

**Methods:**

VAs were conducted in three sites as part of the Improving Methods to Measure Comparable Mortality by Cause (IMMCMC) study: Bohol, Philippines; Chandpur and Comila Districts, Bangladesh; and Central and Eastern Highlands Provinces, Papua New Guinea. Similar diagnostic criteria and cause lists as the Population Health Metrics Research Consortium (PHMRC) study were used to identify gold standard (GS) deaths. We assessed changes in Tariffs by examining the proportion of Tariffs that changed significantly after the addition of the IMMCMC dataset to the PHMRC dataset.

**Results:**

The IMMCMC study added 3512 deaths to the GS VA database (2491 adults, 320 children, and 701 neonates). Chance-corrected cause specific mortality fractions for Tariff improved with the addition of the IMMCMC dataset for adults (+ 5.0%), children (+ 5.8%), and neonates (+ 1.5%). 97.2% of Tariffs did not change significantly after the addition of the IMMCMC dataset.

**Conclusions:**

Tariffs generally remained consistent after adding the IMMCMC dataset. Population level performance of the Tariff method for diagnosing VAs improved marginally for all age groups in the combined dataset. These findings suggest that cause-symptom relationships of Tariff 2.0 might well be robust across different population settings in developing countries. Increasing the total number of GS deaths improves the validity of Tariff and provides a foundation for the validation of other empirical algorithms.

## Background

Reliable knowledge of the distribution of causes of death (COD) in populations is critically important for national and sub-national public health surveillance and planning [1]. However, a substantial proportion of deaths in developing countries occur outside health facilities, leading to low quality COD data [1–4]. Verbal autopsy (VA) has emerged as a cost-effective solution to determining COD in rural areas with limited contact with medical services [5, 6].

VA involves interviewing the family of the deceased to collect information regarding the signs and symptoms surrounding the death. This information can be analyzed by physicians or computer algorithms to assign an individual COD which can then be aggregated to yield population level COD estimates. Currently, most computer algorithms for classifying the COD from verbal autopsy interviews (VAI) rely on a matrix describing the relationship between a set of predictors and a set of causes [7–12]. These associations can be determined either purely empirically, purely through expert opinion, or through a combination of both. Previous studies have shown that methods that rely purely on empirically derived associations, such as random forest and Tariff 2.0, outperform both physician coding and methods in which the associations are derived by expert opinion, such as InterVA [7, 12, 13].

Methods that use empirically derived associations require high quality COD data where the true underlying cause is known as accurately as possible (‘gold standards’) [5]. Automated diagnostic algorithms process data in which the true COD is known with a reasonable degree of certainty to identify predictive patterns in the responses from VAIs. Misclassifications in the underlying COD will likely result in the algorithms learning patterns that are wrong and would result in low-quality COD predictions.

The Population Health Metrics Research Consortium (PHMRC) previously collected VAs matched with COD based on medical record review with strict *ex-ante* diagnostic criteria from six sites in four different countries [14]. Only cases with definitive clinical diagnostic results were included in this study. This ensured that the underlying COD was known with the highest possible degree of certainty. These data were made publicly available in 2013. In this paper, we report on a new study that collected additional gold-standard VAs, adhering to the same strict diagnostic criteria and procedures as for the PHMRC study [15]. These data, collected as part of the Improving Methods to Measure Comparable Mortality by Cause (IMMCMC) study, include gold standard VAs from three sites, two of which were not included in the previous study. We report on the effect of including these data on the stability of the cause-symptom relationship that underlies the Tariff 2.0 diagnostic method.

## Methods

### Data

The original PHMRC study sites included Andhra Pradesh, India; Bohol, Philippines; Dar es Salaam, Tanzania; Mexico City, Mexico; Pemba Island, Tanzania; and Uttar Pradesh, India [14]. In total, 7836 adults, 2075 children, 1629 neonates, and 1002 stillbirths were collected. The study attempted to gather a similar number of cases (at least 100) for each major COD (as reflected in the Global Burden of Disease Study) in representative low and middle income country sites.

The IMMCMC study gathered gold standard VAs between 2011 and 2014 using the PHMRC long form VAI. Cases were identified using the same diagnostic criteria and cause list as the PHMRC study. The IMMCMC study was conducted at three sites: Chandpur and Comilla Districts in Bangladesh, Central and Eastern Highlands Provinces in Papua New Guinea, and Bohol Province in the Philippines. The data collection methodology is described in Additional file 1. Gold standards have been classified as having attained level 1, 2A, 2B, or 3 level of certainty, depending on the amount of information contained in the medical records [14]. Levels 1 and 2 represent cases where the certainty about the diagnosis of the underlying COD is greatest. In this report, we only analyze the 3512 cases that met gold standard level 1 or 2 criteria. This included 2491 adults, 320 children and 701 neonates. The majority of the cases were from Bohol, Philippines, which contributed 2384 cases; 1070 VAs were from Bangladesh and 58 were from Papua New Guinea.

The cause composition of the IMMCMC dataset is less balanced than the PHMRC data because the IMMCMC gathered all deaths at the study sites while the PHMRC study attempted to gather at least 100 cases for pre-determined causes. Table 1 shows the number of cases in each dataset by age module and cause. The IMMCMC database contributed a substantial number of additional deaths due to stroke, pneumonia, acute myocardial infarction, road traffic accidents, stillbirth, and preterm delivery.

**Table 1:** Number of cases by cause and age module for the PHMRC and IMMCMC datasets

	PHMRC		IMMCMC		Combined	
	N	%	N	%	N	%
Adult						
Stroke	630	8.0%	691	27.7%	1321	12.8%
Other Non-communicable Diseases	598	7.6%	275	11.0%	873	8.5%
Pneumonia	539	6.9%	243	9.8%	782	7.6%
Acute Myocardial Infarction	400	5.1%	192	7.7%	592	5.7%
Maternal	467	6.0%	72	2.9%	539	5.2%
AIDS	501	6.4%	8	0.3%	509	4.9%
Other Cardiovascular Diseases	416	5.3%	79	3.2%	495	4.8%
Diabetes	414	5.3%	70	2.8%	484	4.7%
Renal Failure	413	5.3%	62	2.5%	475	4.6%
Road Traffic	202	2.6%	186	7.5%	388	3.8%
Other Infectious Diseases	263	3.4%	97	3.9%	360	3.5%
Cirrhosis	313	4.0%	29	1.2%	342	3.3%
TB	275	3.5%	55	2.2%	330	3.2%
Diarrhea/Dysentery	228	2.9%	27	1.1%	255	2.5%
Falls	173	2.2%	57	2.3%	230	2.2%
COPD	171	2.2%	45	1.8%	216	2.1%
Homicide	167	2.1%	46	1.8%	213	2.1%
Breast Cancer	194	2.5%	9	0.4%	203	2.0%
Suicide	124	1.6%	53	2.1%	177	1.7%
Leukemia/Lymphomas	155	2.0%	18	0.7%	173	1.7%
Cervical Cancer	155	2.0%	3	0.1%	158	1.5%
Poisonings	86	1.1%	57	2.3%	143	1.4%
Other Injuries	103	1.3%	37	1.5%	140	1.4%
Fires	122	1.6%	11	0.4%	133	1.3%
Colorectal Cancer	99	1.3%	23	0.9%	122	1.2%
Lung Cancer	106	1.4%	13	0.5%	119	1.2%
Drowning	106	1.4%	1	0.0%	107	1.0%
Malaria	100	1.3%	0	0.0%	100	1.0%
Stomach Cancer	62	0.8%	11	0.4%	73	0.7%
Bite of Venomous Animal	66	0.8%	3	0.1%	69	0.7%
Asthma	47	0.6%	11	0.4%	58	0.6%
Prostate Cancer	48	0.6%	2	0.1%	50	0.5%
Epilepsy	48	0.6%	1	0.0%	49	0.5%
Esophageal Cancer	40	0.5%	4	0.2%	44	0.4%
Child						
Pneumonia	531	25.7%	124	38.8%	655	27.5%
Diarrhea/Dysentery	256	12.4%	38	11.9%	294	12.3%
Other Defined Causes of Child Deaths	194	9.4%	43	13.4%	237	9.9%
Sepsis	138	6.7%	11	3.4%	149	6.3%
Malaria	116	5.6%	0	0.0%	116	4.9%
Road Traffic	92	4.5%	10	3.1%	102	4.3%
Other Cardiovascular Diseases	76	3.7%	9	2.8%	85	3.6%
Drowning	83	4.0%	1	0.3%	84	3.5%
Other Infectious Diseases	67	3.2%	10	3.1%	77	3.2%
Fires	68	3.3%	3	0.9%	71	3.0%
Meningitis	58	2.8%	12	3.8%	70	2.9%
Hemorrhagic fever	51	2.5%	17	5.3%	68	2.9%
Other Digestive Diseases	48	2.3%	16	5.0%	64	2.7%
Falls	49	2.4%	10	3.1%	59	2.5%
Bite of Venomous Animal	54	2.6%	1	0.3%	55	2.3%
Violent Death	52	2.5%	0	0.0%	52	2.2%
Encephalitis	41	2.0%	1	0.3%	42	1.8%
Other Cancers	28	1.4%	8	2.5%	36	1.5%
Poisonings	18	0.9%	6	1.9%	24	1.0%
Measles	23	1.1%	0	0.0%	23	1.0%
AIDS	20	1.0%	0	0.0%	20	0.8%
Neonate						
Stillbirth	1002	38.3%	370	52.7%	1372	41.3%
Preterm Delivery	659	25.2%	172	24.5%	831	25.0%
Birth asphyxia	461	17.6%	88	12.5%	549	16.5%
Congenital malformation	248	9.5%	33	4.7%	281	8.5%
Meningitis/Sepsis	165	6.3%	32	4.6%	197	5.9%
Pneumonia	82	3.1%	6	0.9%	88	2.7%

### Tariff Method

Tariff 2.0 is based on the determination of a set of Tariffs, based on a matrix of cause-symptom pair endorsement rates [16]. A cause-symptom pair is endorsed if the interviewee responded “yes” to a particular question, for which the true COD was known, or reports a duration greater than a pre-specified cutoff on questions that ask about length of time. Other values, including “Don’t Know”, are considered unendorsed and are counted in the denominator of the endorsement rate. The Tariff for any cause-symptom pair is calculated from the endorsement rate of that given cause-symptom pair and the distribution of endorsement rates for the symptom across all causes. Specifically, it is:

$$ \mathrm{Tarif}{\mathrm{f}}_{i,j}=\frac{x_{i,j}-{\mathrm{Median}}_i}{\mathrm{IQ}{\mathrm{R}}_i} $$

where *x*_*i,j*_ is the endorsement rate for symptom *i* across all cases in which the true cause was *j*, Median_*i*_ is the median endorsement rate of symptom *i* across all causes, and IQR_*i*_ is the interquartile range of endorsement rates of symptom *i* across all causes. For example, if 50% of respondents answered “yes” to chest pain for Acute Myocardial Infarction, and the median and interquartile range for chest pain across all causes were 18 and 20%, respectively, then the Tariff for chest pain for Acute Myocardial Infarction my would be 1.6.

Tariffs are then tested for significance using a Monte Carlo experiment. The original symptom data is resampled with replacement, stratified by cause, to create 500 datasets. In each dataset, the total number of observations for each cause is constant, but the given rows, and thus the endorsements and endorsement rate, varies. Tariffs are calculated from each of these datasets and are then used to create 99% uncertainty intervals around each Tariff estimate. Tariffs where the uncertainty interval includes zeros, i.e. where we are not certain about the directionality of the association between the symptom and the cause, are removed. Lastly, Tariffs are rounded to the nearest 0.5. This prevents over-fitting by treating similar values as containing the same amount of information instead of prioritizing minor, insignificant difference in predictive value.

### Analysis

We assessed changes in the Tariffs by comparing the proportion of significant Tariffs before and after the addition of the IMMCMC dataset. We also measured the proportion of Tariffs that changed significantly or changed directionality (i.e. from positive to negative). We performed the out-of-sample validation procedure described in Murray et al. to assess changes in individual and population-level COD performance [17]. Individual-level performance was assessed using chance-corrected concordance (CCC). CCC is a measure of agreement between the predicted and gold standard cause assignment, adjusted for chance. Population-level performance was assessed using cause-specific mortality fraction (CSMF) accuracy and chance-corrected cause-specific mortality fraction (CCCSMF) accuracy [18]. CSMF accuracy is a summary measure of performance between the predicted and gold standard cause assignment, and CCCSMF adjusts for chance.

## Results

Adding the IMMCMC dataset did not change the directionality of the association between any cause-symptom pairs. In other words, the sign of all 2852 significant Tariffs was the same before and after adding the new data.

In the original study using only PHMRC data, 2852 of the 19,401 (14.7%) cause-symptom pairs across the three modules were statistically significant. After combining the PHMRC and IMMCMC datasets and recalculating Tariffs, 2563 of the original 2852 (89.9%) values remained significant. 97.2% of Tariffs did not change significantly after the addition of the IMMCMC dataset; less than 3% did.

Table 2 shows the cause-specific change in performance measured by CCC, with individual causes ranked in decreasing order according to the magnitude of the difference in CCC before and after the addition of the new cases. For adults, CCC was highest for injuries and lowest for residual categories for both the PHMRC and combined datasets. For children, CCC was highest for injuries and lowest for infectious diseases and residual categories for both the PHMRC and combined datasets. For neonates, CCC was much higher for stillbirth than all other causes in both datasets. CCC for pneumonia and birth asphyxia were low for both datasets. In short, addition of the new cases did not alter the comparative performance of the Tariff method for various causes of death, as measured by CCC. Interestingly, nearly all adult and child causes experienced an increase in CCC, with the largest decrease for other injuries. Neonate causes mainly experienced an increase or little change, except for a decrease in meningitis/sepsis.

**Table 2:** Changes in Cause-Specific chance corrected concordance (CCC) with additional Gold Standard cases

	Median Chance-Corrected Concordance (%)				
	PHMRC	95% UI	Combined	95% UI	Difference
Adult Cause					
Fires	68.8	(67.4, 71.1)	77	(75.7, 78.1)	8.2
Leukemia/Lymphomas	27.4	(25.6, 28.4)	34.7	(33.6, 35.7)	7.3
Esophageal Cancer	59.6	(58.1, 61.9)	65.6	(63.1, 67.1)	6
Road Traffic	82.8	(82.1, 83.7)	87.4	(86.6, 88.1)	4.6
AIDS	50.5	(49.6, 51.6)	54.9	(53.8, 55.8)	4.4
Other Infectious Diseases	12.6	(11.7, 13.5)	16.8	(16.1, 17.5)	4.2
Maternal	80.8	(80, 81.9)	84.8	(83.9, 85.3)	4
Renal Failure	34.1	(33.1, 35.5)	38.1	(37.2, 38.6)	4
Bite of Venomous Animal	94.3	(93.5, 95.3)	98.2	(96.2, 100)	3.9
Pneumonia	15.5	(14.3, 16.3)	19.3	(18.8, 20)	3.8
Stroke	55.7	(54.3, 56.3)	59.1	(58.4, 60.1)	3.4
Homicide	77.9	(77, 78.9)	81.2	(79.8, 81.9)	3.3
Other Cardiovascular Diseases	25.9	(24.4, 27.2)	29	(28.1, 30.1)	3.1
Falls	60.9	(59.9, 62.3)	63.9	(62.8, 64.5)	3
Diarrhea/Dysentery	36.5	(35.5, 37.9)	39.2	(38.1, 40.3)	2.7
Prostate Cancer	60.6	(58.8, 62.5)	63	(60.7, 64.7)	2.4
Diabetes	43.3	(42.2, 44.7)	45.6	(44.8, 46.4)	2.3
Lung Cancer	24.3	(22.7, 25.8)	26.1	(24.7, 27.4)	1.8
Malaria	45.7	(43.4, 46.8)	47.5	(46, 48.4)	1.8
Ischemic Heart Disease	32.7	(31.6, 33.7)	34.4	(33.2, 35.2)	1.7
Other Cancers	5.3	(4.4, 5.8)	6.9	(6.2, 7.3)	1.6
Cervical Cancer	66.9	(65.6, 67.8)	67.9	(66.4, 69.3)	1
Colorectal Cancer	22.5	(20.6, 23.6)	23.5	(22.2, 24.7)	1
Other Non-communicable Diseases	10.3	(9.7, 10.9)	11.2	(10.5, 11.6)	0.9
Breast Cancer	78.7	(77.7, 79.8)	79.4	(78.5, 80.1)	0.7
Poisonings	57	(55.8, 58.8)	57.6	(55.9, 59)	0.6
Chronic Respiratory	45.3	(44.2, 46.3)	45.6	(44.5, 46.7)	0.3
Drowning	89.3	(88.3, 90.1)	89.5	(88.5, 90.6)	0.2
Stomach Cancer	26.2	(24, 27.5)	23.5	(22.1, 24.8)	−2.7
Cirrhosis	51.5	(49.7, 52.8)	48.4	(48.4, 50.2)	−3.1
TB	45.8	(44.6, 47.2)	38.5	(37.8, 39.8)	−7.3
Suicide	13.6	(12.3, 14.2)	6	(5.3, 6.6)	−7.6
Other Injuries	64.6	(63.1, 65.6)	52.3	(50.9, 53.9)	−12.3
Child Cause					
Falls	55.6	(52.7, 58)	66.7	(64.9, 69.6)	11.1
Other Cancers	31.7	(29.4, 34.1)	42.5	(39.7, 45)	10.8
Pneumonia	10	(9.3, 11.2)	16.6	(15.4, 17.9)	6.6
Fires	65.4	(63.9, 68.6)	71.7	(69.9, 73.8)	6.3
Other Digestive Diseases	24.5	(21.3, 25.7)	29.9	(27.5, 31.1)	5.4
Other Cardiovascular Diseases	36.8	(35, 38.6)	40.3	(38.9, 42.4)	3.5
Measles	80.6	(78.1, 83.6)	83.5	(81.2, 85)	2.9
Poisonings	68.9	(65, 71.6)	71.8	(68.8, 73.8)	2.9
Road Traffic	92.7	(91.3, 93.8)	95.4	(93.8, 96.3)	2.7
Sepsis	6.4	(5.3, 7.9)	8.8	(7.4, 10)	2.4
Malaria	56.3	(54.9, 58)	58	(56.8, 60.5)	1.7
Bite of Venomous Animal	100	−100,100	100	(97.2, 100)	0
Diarrhea/Dysentery	36.1	(34.2, 37)	36	(34.4, 37.8)	−0.1
Encephalitis	30	(29.6, 33.5)	29.8	(27.1, 31.8)	−0.2
Drowning	94.7	(93.1, 96)	94.5	(93.4, 95.5)	−0.2
Violent Death	85.2	(83.5, 87.2)	85	(83.4, 86.8)	−0.2
Hemorrhagic fever	55.8	(52.8, 58)	55	(53.2, 57)	−0.8
Other Defined Causes of Child Deaths	17.5	(16.4, 19)	15.8	(14.1, 17.5)	−1.7
AIDS	50.8	(47.4, 53.7)	47.5	(47.2, 52.9)	−3.3
Meningitis	20.3	(17.9, 22.2)	15.7	(13.4, 16.9)	−4.6
Neonate Cause					
Other Infectious Diseases	22.4	(21.1, 25)	17.6	(16, 19.2)	−4.8
Birth asphyxia	32.8	(31.6, 33.6)	35.9	(34.9, 36.9)	3.1
Preterm Delivery	43.2	(42.1, 44.1)	45.8	(44.9, 46.7)	2.6
Pneumonia	13.4	(12, 14.6)	14.2	(12.6, 15.1)	0.8
Stillbirth	88.7	(88, 89.2)	88	(87.5, 88.5)	−0.7
Congenital malformation	36.2	(35.4, 37.2)	35	(33.7, 36.2)	−1.2
Meningitis/Sepsis	38.4	(36.9, 40)	32.2	(31, 33.3)	−6.2

Abbreviations: *UI* Uncertainty Interval

While the changes in Tariffs with the addition of new data were generally small, it is important to understand for which cause-symptom pairs the incorporation of new data had greatest effect. Table 3 shows the ten largest increases and decreases in Tariffs (all Tariffs shown in Additional file 2). Tariffs represent the strength (positive or negative) of the relationship between a particular cause and a given symptom, so large increases in Tariffs indicate increased importance of that symptom for the given cause, while large decreases signify the opposite. The largest changes in Tariffs were mainly associated with injury and maternal deaths where Tariffs would expect to be high because of the likelihood that symptoms for these conditions would be more clearly distinguishable and remembered. All symptoms associated with large increases in Tariffs had a strong association with a particular cause. The same pattern was also observed for large decreases. Although the absolute change in Tariffs for these pairs might have been large, the strength of the association was sufficiently clear that the change did not distort the predictive ability of the algorithm in selecting the correct underlying COD.

**Table 3:** Ten largest changes (increases or decreases) in Tariff values after incorporation of additional gold standard data

Cause	Module	Symptom	Tariff score based solely on PHMRC	Tariff score based on Combined	Difference
Bite of Venomous Animal	adult	Decedent suffered bite/sting	229.7	327.9	98.2
Fires	adult	Decedent suffered burn	178.3	241.3	63.0
Fires	child	Decedent suffered burn/fire	125.5	156.9	31.4
Maternal	adult	Did she have excessive bleeding during labor or delivery?	73.5	87.0	13.4
Suicide	adult	Decedent suffered poisoning	127.5	140.4	12.9
Breast Cancer	adult	Did [name] have any swelling or lump in the breast?	39.2	49.1	9.9
AIDS	child	Was the HIV test ever positive?	57.6	66.2	8.6
Breast Cancer	adult	Did [name] have any ulcers (pits) in the breast?	65.5	72.5	7.1
Falls	adult	Decedent suffered fall	33.6	40.7	7.0
AIDS	adult	Did Decedent Have AIDS?	16.7	23.5	6.9
AIDS	adult	Did Decedent Have TB?	13.9	10.0	−3.9
Maternal	adult	For how many months was she pregnant? [days]	279.4	275.4	−4.0
TB	adult	Did Decedent Have TB?	16.7	11.4	−5.3
Drowning	adult	Decedent suffered drowning	839.6	831.8	−7.8
Other Non-communicable Diseases	adult	For how long before death did the convulsions last? [days]	22.3	14.3	−8.0
AIDS	child	Has the deceased’s (biological) mother ever been told she had AIDS by a health worker?	41.4	31.5	−9.9
Maternal	adult	Was [name] pregnant at the time of death?	36.6	25.7	−10.9
Stroke	adult	Paralyzed upper part of body	25.4	12.1	−13.3
Maternal	adult	Did she die within 6 weeks after having an abortion?	40.5	26.0	−14.5
Poisonings	adult	Decedent suffered poisoning	384.4	254.6	− 129.9

Note: Symptoms include non-word symptoms where the change in Tariff value was at least 1.0 and the Tariff value using either the PHMRC dataset or the combined PHMRC/IMMCMC dataset is at least 1.0. Tariff values of zero represent Tariffs that are not statistically significant

This conclusion is confirmed by Table 4 which shows the overall change in predictive performance for Tariff 2.0 before and after the IMMCMC dataset was added to the PHMRC dataset (full performance details are shown in Additional file 3). In fact, overall diagnostic predictive accuracy for adults and children increased marginally for both populations (CSMF) and individuals (CCC), and for neonatal CSMFs, but decreased slightly when assessing diagnostic accuracy for individual neonatal deaths (CCC).

**Table 4:** Average change in overall diagnostic performance before and after incorporation of the IMMCMC data set (using the PHMRC Shortened Questionnaire)

	Adult	Child	Neonate
CCC	+ 1.2%	+ 5.6%	−1.5%
CSMF Accuracy	+ 0.8%	+ 1.0%	+ 0.3%
CCCSMF Accuracy	+ 5.0%	+ 5.8%	+ 1.5%

## Discussion

Automated diagnostic methods such as Tariff 2.0 have the potential to revolutionize national mortality surveillance system by facilitating huge improvements in the availability and quality of data on causes of death in hitherto underserved populations. But are these methods reliable and generalizable and likely to perform similarly in different populations? This study has confirmed the robustness of the Tariff 2.0 method when new gold-standard data from different populations were incorporated. The addition of the IMMCMC data to the publicly available PHMRC dataset confirmed the results of the original Tariffs derived solely from the PHMRC dataset, and led to a slight overall improvement in the diagnostic performance of the algorithm.

Adding additional deaths to the PHMRC dataset to calculate Tariffs further clarified the relationship between various symptoms and causes. No Tariffs changed direction (i.e. went from positive to negative, or the converse) and the vast majority of Tariffs that were significant using the PHMRC dataset were also significant when using the combined dataset. Some Tariffs which were statistically significant in the original PHMRC data were not, when using the combined dataset. These differences likely reflect instances where the Tariffs were over-fit to ‘noise’ in the raw data, and the addition of new data served to create more generalizable Tariffs.

Given that the majority of causes experienced an increase in CCC, most changes in the cause-symptom relationship as a result of adding new data led to improved predictive performance. Decreases in the CCC for some causes were likely due to spurious associations between symptom and cause that were a result of relatively few deaths present in both the PHMRC and IMMCMC datasets for certain causes. For example, only 6 neonatal pneumonia deaths (7% increase) and 32 meningitis/sepsis deaths (19% increase) were added to the PHMRC dataset from the IMMCMC study. Rather, 80–90% of neonatal deaths were attributed to stillbirth, preterm delivery, or birth asphyxia. The similar symptoms of pneumonia and meningitis/sepsis, together with the comparatively few cases, provided insufficient information for Tariff to distinguish between the causes, resulting in a decrease in CCC for neonates. A greater number of deaths attributed to more causes in the adult and child modules contributed to the increased diagnostic performance of Tariff when applied to the combined dataset.

Large changes in the Tariffs tended to be limited to certain maternal and injury causes. These changes reflect the addition of a diversity of new cases that were not in the PHMRC dataset and suggest the likelihood of cultural differences affecting responses to some questions pertinent to maternal and injury deaths. Difficulties with describing the intent of the question, or communication skills, are also likely to affect the quality of the interview process. Given the multiple requirements of interviewers when conducting a VAI, it is hardly surprising that incomplete or misleading data will be collected in some cases. This makes it much harder for the algorithm to correctly predict the most probable COD, and likely lead to unsubstantiated changes in Tariffs.

The diversity of study populations present in the combined dataset suggests that the associations generated by the algorithm are likely to be generalizable. Otherwise, the associations may reflect a cultural bias in which symptoms are noticed, communicated, remembered and reported from a limited set of study populations. Previous studies have shown that respondents often report different information at repeat visits regarding the same death, but key symptoms are often remembered and are sufficient to properly classify the COD [19, 20]. With a large enough training data set, algorithms should be able to distinguish between these key predictors and background noise. It is also necessary to include VAs which include missing data or where the pattern of responses may not seem consistent with the true COD, as long as they were collected under real survey conditions. These observations represent ‘noise’ in the data that are propagated when the algorithm is applied to deaths notified to vital registration systems. Properly calibrated computer algorithms such as Tariff 2.0 will be able to account for this ‘noise’ and adjust the predictions accordingly.

The addition of deaths from the IMMCMC study to the PHMRC GS database is an important step in the continual validation of empirical VA algorithms. There has been some criticism of empirical methods that are derived and tested on gold standard datasets, but it is important to recognize the benefits of a GS database [9, 21–23]. First, GS deaths provide evidence that the responses provided during a VA do not necessarily make sense in a clinical context. For example, 147 deaths in the PHMRC dataset were reported as stillbirth but described as neonatal deaths, which is impossible because stillbirth implies the birth did not occur [21]. Second, GS deaths provide a basis for assessing the validity for text items in open-ended responses. The potential of these “open narrative” responses to improve diagnostic accuracy has yet to be fully realized [16]. Third, deaths that occur in-hospital are different than deaths that occur at home because the terminal events are prolonged by therapeutic activity, but the signs and symptoms which precipitated the hospital admission are what are asked in a VA [9]. Some diseases may have different presentations at home than in the hospital: e.g. families generally have much less chance to observe a woman dying in labor or a neonate dying in a special nursery in a hospital than they would have of observing these events at home. Collecting GS data for such conditions would set standards for such data collection environments. While GS databases have limitations, they provide a valuable basis for VA validation research and implementation. They also set the foundation for adding additional cases of deaths that can provide empirical evidence about the generalizability of VA methods.

We have categorized the IMMCMC dataset as gold standard, but we recognize its limitations. The sampling strategy of collecting deaths in the IMMCMC study was different from that of the PHMRC study. All deaths in study hospitals were collected for the IMMCMC dataset, while approximately 100 deaths per cause were intended for collection in the PHMRC dataset. This difference may bias the cause-symptom relationship of less frequent causes in the IMMCMC dataset towards that of the PHMRC dataset. Furthermore, while the IMMCMC dataset added 3513 death cases, some causes (e.g. AIDS and lung cancer) had less than 20 deaths. Changes in the Tariffs for these causes may simply reflect noise. Last, the additional death cases from the IMMCMC sites occurred in one of the same sites as the PHMRC sites (Bohol, Philippines), so the results of the combined database are not as generalizable to the rest of the world had the deaths come from regions that are not present in either dataset, such as South America, or have low representation, such as Africa; they do however, support broader generalizability in Asia and the Pacific.

## Conclusions

Additional observations for training data are useful for refining the association between symptoms and causes. While the original dataset collected in the PHRMC study is sufficiently large to derive confidence in the Tariffs for most symptom-cause pairs that underlie the Tariff diagnostic method, adding new data further clarifies the complex associations between symptoms and causes reported during a VA interview. The addition of the IMMCMC dataset to the PHMRC database increased the cause-specific performance metrics for most causes and overall performance increased for adults, children, and neonates, at least at the population level. Including new observations changed the Tariffs of some key symptoms, which may indicate cultural differences in respondents or noisy data, but overall the inclusion of new data did not alter previous findings about the diagnostic accuracy of the Tariff method for VAs, nor its predictive performance. While the findings of this study suggest that the Tariffs are relatively invariant to cultural differences in respondent populations, this needs to be more firmly established on the basis of a large dataset of gold standard cases from a wide variety of locations. This is a priority for VA research, particularly as the method is gaining increasing popularity for widespread use in vital registration systems.

## Supplementary information

**Additional file 1.** Improving Methods to Measure Comparable Mortality by Cause Study Sites. Description of data collection sites in the Philippines, Bangladesh, and Papua New Guinea.

**Additional file 2.** Tariffs and Endorsements Rates by Cause, Symptom, and Site. Tariffs and endorsement rates with confidence intervals for each cause and symptom at all study sites from the IMMCMC and PHMRC studies. (CSV 3651 kb)

**Additional file 3.** Tariff 2.0 Performance Metrics. CCC, CSMF accuracy, CCCSMF accuracy, and cause predictions across all 500 Dirichlet splits for each age module and study.

## Data Availability

All data generated or analysed during this study are included in this published article and its supplementary information files.

## Abbreviations

CCC: Chance-corrected concordance
CCCSMF: Chance-corrected cause-specific mortality fraction
COD: Cause of death
CSMFs: Cause-specific mortality fractions
IMMCMC: Improving Methods to Measure Comparable Mortality by Caus
PHMRC: Population Health Metrics Research Consortium
VA: Verbal autopsy
VAI: Verbal autopsy instrument
