# Supplementary material for: Robustness of the Tariff method for diagnosing verbal autopsies: impact of additional site data on the relationship between symptom and cause
Source: BMC Med Res Methodol. 2019 Dec 9;19:232. doi: 10.1186/s12874-019-0877-7 (PMC6905113; doi:10.1186/s12874-019-0877-7)
Supplement: Supplementary file 1 — Additional file 1. Improving Methods to Measure Comparable Mortality by Cause Study Sites. Description of data collection sites in the Philippines, Bangladesh, and Papua New Guinea. [file 12874_2019_877_MOESM1_ESM.docx]

Bohol, Philippines is an island province with a population of about 1.2 million. It has 47 municipalities and one city (Tagbilaran City). Verbal Autopsies were collected from all deaths in 11 of the municipalities which had been selected as clusters with probability of selection proportional to size. Deaths were identified by the capture-recapture method from three sources: the civil register, health center records, and the Catholic Church parish registers.

The Matlab Subdistrict in Chandpur District of Bangladesh has a total population of approximately 225,000. Verbal Autopsies were collected from all deaths in the Matlab Health and Demographic Surveillance System (HDSS).

Deaths in Papua New Guinea were identified from the Partnerships in Health HDSS in four sites: Hiri, Central Province; Hides, Southern Highlands Province; Asaro Valley, Eastern Highlands Province; Karkar, Madang Province.
